# Antiobesity Effects of Anthocyanins in Preclinical and Clinical Studies

**DOI:** 10.1155/2017/2740364

**Published:** 2017-07-13

**Authors:** Elena Azzini, Jasminka Giacometti, Gian Luigi Russo

**Affiliations:** ^1^Council for Agricultural Research and Economics (CREA), Research Center for Food and Nutrition, Via Ardeatina 546, 00178 Rome, Italy; ^2^Department of Biotechnology, University of Rijeka, Radmile Matejčić 2, HR-51000 Rijeka, Croatia; ^3^Institute of Food Sciences, National Research Council, 83100 Avellino, Italy

## Abstract

The natural phytochemicals present in foods, including anthocyanins, might play a role in attenuating obesity by producing a decrease in weight and adipose tissue. This review focused on current knowledge about anthocyanins' role in obesity and its related comorbidities reported in animal models and humans. We summarized their target identification and mechanism of action through several pathways and their final effects on health and well-being. Into consideration of ongoing researches, we highlighted the following key points: a healthy relationship between anthocyanin supplementation and antiobesity effects suffers of the same pros and cons evidenced when the beneficial responses to other phytochemical treatments towards different degenerative diseases have been considered; the different dosage applied in animal versus clinical studies; the complex metabolism and biotransformation to which anthocyanins and phytochemicals are subjected in the intestine and tissues; the possibility that different components present in the supplemented mixtures can interact generating antagonistic, synergistic, or additive effects difficult to predict, and the difference between prevention and therapy. The evolution of the field must seriously consider the need to establish new and adequate cellular and animal models which may, in turn, allow the design of more efficient and prevention-targeted clinical studies.

## 1. Introduction

The obesity and its related comorbidities represent an emerging global health issue. In 2014, WHO estimated overall that about 13% of the world's adult population (11% of men and 15% of women) were obese. Additionally, the worldwide prevalence of obesity is more than doubled between 1980 and 2014 [1]. Obesity is an abnormal excess accumulation of fat in adipose tissue due to an imbalance between energy intake and energy expenditure. The daily energy expenditure consists of basal metabolism, thermogenesis, which includes all processes to produce heat and energy expenditure for physical activity level. The key element to counteract obesity is moderate weight loss (5–10%) over time through an integrated treatment [2]. This is expressed by

- Energy intake: a low-calorie diet providing about 800–1500 kcal/day exerts a slow and progressive weight loss and allows to achieve weight reduction;
- Physical activity: the association between diet with physical activity leads to a greater weight loss but also to an easier weight control compared to only low calorie diet;
- Behavior and lifestyle modification;
- Drug therapy: all drugs act by modulating the neurotransmitter release at neuronal level, regulating, in this way, hunger and appetite.

More recently, natural phytochemicals present in foods, including anthocyanins, due to their chemical structure, seem to be able to exert several pharmacologic activities, mainly antioxidant and anti-inflammatory actions [3]. It has been reported that the anthocyanins might play a role in attenuating obesity by producing a decrease in weight and adipose tissue [4]. Controversial results were obtained in animals and human studies in obesity condition [5–11] by type of supplementation (fresh and/or commercial product or pure compound), dose, length of the study, and by different sizes and clinical characteristics of the enrolled sample. Furthermore, the pharmacological actions of anthocyanins could not be fully recognized without knowing the effects of treatment of anthocyanins alone, the effects of nonanthocyanin molecules, and the possible synergistic action between anthocyanins and the complex mixture of phytochemicals present in whole foods [12]. Thus, this review is focused on the current knowledge about anthocyanins' role in obesity and its related comorbidities reported in animal models and humans.

## 2. Anthocyanins

### 2.1. Anthocyanin Chemistry

**Figure 1:**
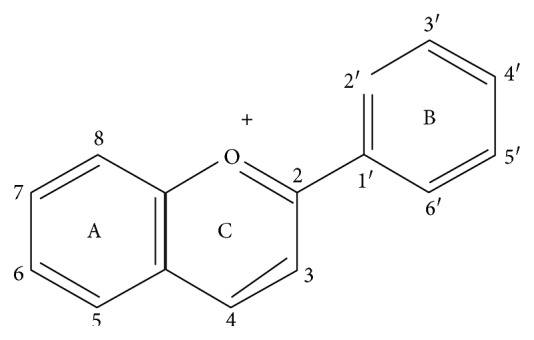
Chemical structures of flavylium cation.

Anthocyanins are water-soluble glycosides of polyhydroxyl and polymethoxyl derivatives of 2-phenylbenzopyrylium or flavylium salts as displayed in Figure 1.

**Figure 2:**
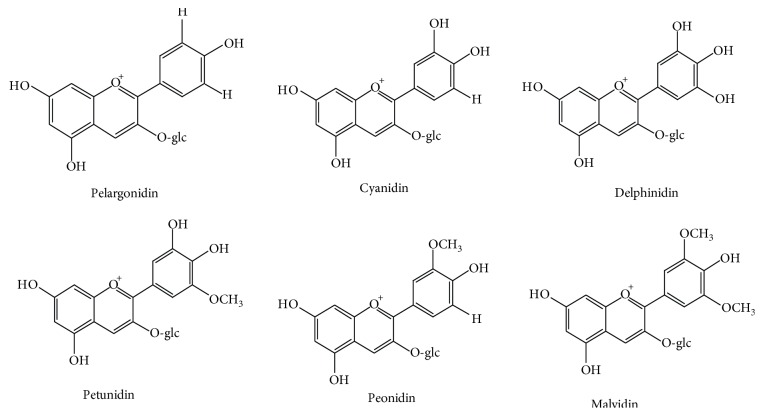
Main six anthocyanidins present in plant foods.

A lot of molecules have been identified [13] by spectrometric measurements, but plant foods are rich in mainly six anthocyanidins (free of sugar usually known as aglycone), including pelargonidin (Pg), cyaniding (Cy), petunidin (Pt), peonidin (Pn), delphinidin (Dp), and malvidin (Mv). Their distribution in fruits and vegetables is Cy 50%, Dp 12%, Pg 12%, Pn 12%, Pt 7%, and Mv 7%. In fact, 90% of anthocyanins are based on cyanidin, delphinidin, and pelargonidin and their methylated derivatives [14]. These compounds are different from each other by the number of hydroxyl substituent groups on the B ring and their methylation degree and by nature, number, and location of sugars attached to the molecule (anthocyanins) (Figure 2) [15].

Generally, the conjugated sugar bonded to carbon 2, 3, and 5 are glucose, arabinose, rhamnose, and galactose. It is possible to distinguish between mono-, di-, and tri-glucosides. Kong et al. [16] have reported the presence of the 3-glucoside derivatives 2.5 more frequent than the 3,5-diglucosides. Glycosylation increases water solubility and stability due to hydrogen bonding; therefore, anthocyanidins rarely occur in their free form due to their high reactivity.

Esterification of sugars represents a key factor for chemical structure of these pigments, and it is based on carboxylic acids, including caffeic, ferulic, sinapic, and the p-coumaric acid and on aliphatic acids as well as acetic acid, the malonic, oxalic, and succinic acid. On the same anthocyanin, it could be present up to three esterifying agents. All these variables contribute to define the unique composition in anthocyanins of foodstuff.

**Figure 3:**
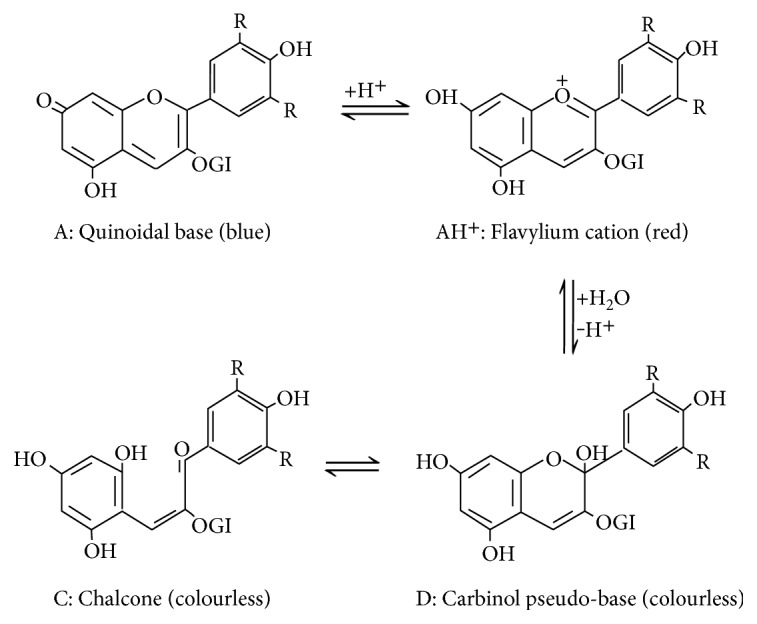
The four chemical forms in equilibrium with each other: the case of Malvidina 3-O-glucoside structures.

The anthocyanins are very stable and highly colored at low pH (pH 1–3). On increasing pH, these molecules change their chemical structure, as well as color. At pH 1–3, the flavylium cation is the predominant species and contributes to purple and red colours. pH 4-5 generates the colorless carbinol. At pH 6-7, quinoidal blue-violet species are dominant, at pH 7-8 the chalcone species, also practically colorless. In the aqueous phase, these four chemical forms are in equilibrium with each other (Figure 3); the prevalent form depends on the pH of the solution. More particularly, the bioavailability of anthocyanins is low due to their sensitivity to changes in pH [17].

### 2.2. Dietary Anthocyanins Source

Plant phenolic fraction is composed by a heterogeneous mixture of molecules belonging to different families with varying chemical structures which content represents a peculiar characteristic of plant tissues. Anthocyanins, belonging to flavonoids family, are water-soluble pigments responsible for most of the red, blue, and purple colors of fruits and vegetables and other plant tissues or products. Anthocyanins, as well as plant phytochemicals, play several and varied functions, but their main activities are to protect plants from oxidative risk posed by various environmental stressors (sunlight and other environmental agents) and to defense plants from fungal, bacterial, or viral infections. Recently, their dietary consumption has been associated to several health benefits by free radical scavenging, antioxidant capacities and antibacterial activity [18, 19]. Nowadays, it does not exist a recommended daily allowance for these molecules although a consumption between 250–400 mg/d, respecting the seasonality of food sources, has been proposed for total flavonoids [20]. European Prospective Investigation into Cancer and Nutrition (EPIC) [21] estimated a total anthocyanidin mean intake of 64·88 (SE 1·86) mg/d and 44·08 (SE 2·45) mg/d for men and women, respectively (Turin, Italy). In plant-derived anthocyanin-rich foods, the relative abundance and composition of anthocyanins differs due to intrinsic and extrinsic factors, such as genetic and agronomic variation, light intensity and type, temperature, harvest time, storage, and processing condition. Additionally, data on food anthocyanins composition and content are limited and debated [22]. Zamora-Ros et al. [20] have reported a dietary data set in combination with anthocyanidins content data from two databases USDA database for the flavonoid content of selected foods [23] and Phenol-Explorer [24]. This database includes 1877 food items; the anthocyanidins are calculated as the sum of the available forms (glycosides and aglycones) in the literature and expressed as anthocyanidin aglycones per 100 mg fresh weight. To exert their bioactivities and so to enhance health benefits, the anthocyanins should be absorbed at useful dose and properly metabolized to reach specific organ target. More particularly, the bioavailability of anthocyanins is low due to their sensitivity to changes in pH.

### 2.3. Anthocyanin Bioavailability

**Figure 4:**
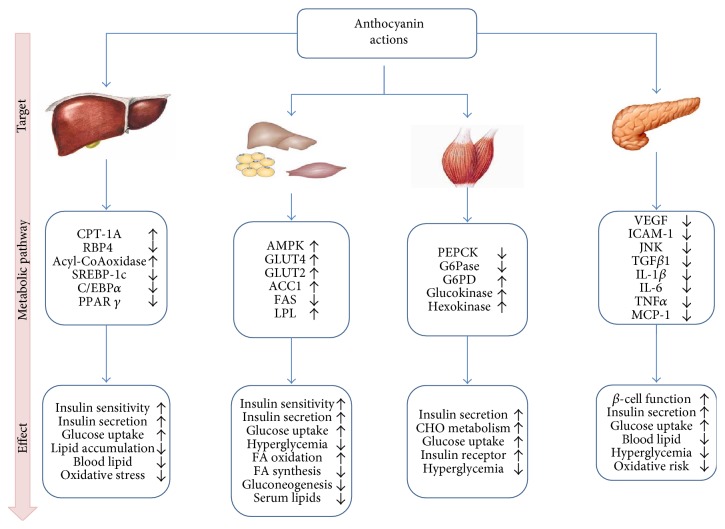
Proposed anthocyanins target identification and mechanism-of-action through several pathways and their final effects on health and well-being. CPT1A: carnitine palmitoyltransferase-1A; RBP4: retinol binding protein-4; SREBP1c: sterol regulatory element binding protein-1c; C/EBP*α*: CCAAT enhancer binding protein-*α*; PPAR *γ*: peroxisomal proliferator-activated receptor gamma; AMPK: 5′ adenosine monophosphate-activated protein kinase; GLUT4: glucose transporter 4; GLUT2: glucose transporter 2; ACC1: acetyl-CoA carboxylase; FAS: fatty acid synthase; LPL: lipoprotein lipase; PEPCK: phosphoenolpyruvate carboxykinase; G6pase: glucose-6-phosphatase; G6PDH: glucose-6-phosphate dehydrogenase; VEGF: vascular endothelial growth factor; ICAM-1: intercellular adhesion molecule; JNK: c-Jun N-terminal kinase; TGF*β*1: transforming growth factor-beta; IL-6: interleukin-6; TNF-*α*: tumor necrosis factor alpha; MCP-1: monocyte-chemo-attractant protein-1.

Anthocyanins are generally stable at pH values of 3.5 or below, therefore stable under stomach conditions (pH 2). However, these molecules are degraded quickly at higher pH values, such as the intestinal tract (pH 7), so the beneficial absorption and nutritional value could be significantly reduced. Different effects have been observed after acute and chronic consumption of anthocyanin-rich foods or their extracts demonstrating the high interindividual bioavailability and bioactivity [25, 26]. Several metabolic pathways, tissue distribution, and effects have been reviewed [27, 28]. The absorption, metabolism, and excretion of anthocyanins depend on food matrix, including other antioxidants and macronutrients present in the usual diet, which consequently affects anthocyanin bioavailability. The pharmacokinetics of anthocyanins and metabolites from several foods suggest that the gastrointestinal tract is their primary target [29–31]. Fang [32] summarized how some anthocyanins could be efficiently absorbed from the gastrointestinal lumen, undergo extensive first-pass metabolism, and enter the systemic circulation as metabolites. Nevertheless, the production of several metabolites poorly absorbed and rapidly removed from plasma does not impair to ameliorate obesity and related comorbidities [33, 34]. Furthermore, Cardona et al. [35] reviewed that, like drugs and/or xenobiotics, these molecules are processed in the body by enzymatic activities of the gut microbial community. On releasing, their metabolites contribute to the maintenance of gut health by the modulation of the gut microbial balance through the stimulation of the growth of beneficial bacteria and the inhibition of pathogen bacteria, exerting prebiotic-like effects. The interindividual differences in the composition of the microbiota could contribute to differences in bioavailability and bioefficacy of anthocyanins and their metabolites. Moreover, Smeriglio et al. [36] highlighted the influence of human enzymes polymorphism involved in biotrasformation mechanisms on anthocyanins bioavailability. Finally, studies on the gut microbiota modulation by anthocyanins and their mechanisms are incomplete. In Figure 4(), we summarized anthocyanin's target identification and mechanism of action through several pathways and their final effects on health and well-being. Briefly, they act on the skeletal muscle, liver, pancreas, and adipose tissue for stimulating different inflammatory cytokines, metabolic enzymes, and signaling pathways, which exert anti-inflammatory, antioxidative, and metabolic-stabilizing activity. These mechanisms are associated with stabilization of obesity and diabetes, improvements in blood pressure and lipid profiles, decreased atherosclerotic development, and improved vascular function.

## 3. The Role of Anthocyanins in Obesity: Animal Studies

In the last several years, many research groups carried out studies to identify the link between anthocyanin compounds and hyperlipidemia, hyperglycemia, hypertension, inflammation, and immunity that cause diabetes, cardiovascular diseases, and other inflammation-related diseases. Obesity is one of public health problems because it leads to the development of metabolic disorders. Anthocyanins are known as compounds that can modulate mechanisms of the homeostasis of glucose, lipids, and amino acids and suppress inflammation. Increasing attention has been given to the development of alternative strategies and possible therapies targeting differentiation of adipogenesis, glucose transport and intake, attenuation of inflammation, and changes in the immune response. During long-term and low-level inflammation, usually present in obese subjects, alterations presented in the metabolism could lead to changed immunity [37].

Understanding the effects of anthocyanins on human health and disease prevention could promote interest in drug discovery and the potential of diets in the prevention of obesity and several diseases.

Prior et al. [38] reported that supplemented diet with a blueberry extract (BBE) significantly decreased body weight and body fat accumulation in obese C57BL/6 mice fed with high-fat (HF) diet, while intake of wild blueberry powder (WBP) did not induce body fat accumulation. The same authors, in the following study [26], reported that consumption of blueberry juice (BBJ) did not reduce body weight and the weight of white adipose tissue (epididymal and retroperitoneal fat) in obese mice [26, 39]. A fermented blueberry-blackberry beverage also mitigates the development of obesity and reduces fasting blood glucose in C57BL/6J mice [40]. In addition, the consumption of both BBJ and mulberry juice (MJ) resulted in a decrease of body weight, decrease of the serum cholesterol, and reduced resistance to insulin (IR) as well as reduced lipid accumulation and decreased leptin secretion [41].

Beneficial effects of BB anthocyanins reflect the ability of BB anthocyanins to change mitogen-activated protein kinase (MAPK) and nuclear factor-*κ*B (NF-*κ*B) stress signaling pathways, which might suggest their cytoprotective and anti-inflammatory role in the pathology of obesity [39].

Seymour et al. [42] described that supplementation with 2% (wt/wt) blueberry powder (BBP) reduced the weight of intraperitoneal fat and enhanced the activity of the peroxisome proliferator-activated receptor (PPAR) in white adipose tissue (WAT) and skeletal muscle in Zucker-fatty rats [42]. Following the above mentioned studies, Vendrame et al. [43] reported that supplementation with 8% (wt/wt) wild blueberry powder (WBP) after 8 weeks of feeding significantly increased blood adiponectin levels as well as reduced the levels of inflammatory markers in WAT [43] and improved dyslipidemia [6]. However, WBP did not obtain reduced fasting blood glucose (FBG) and insulin levels in obese Zucker rats in this study [44].

Bilberries can also reduce inflammation, prevent chronic hypertension, and thus mitigate the development of obesity as reported by Mykkänen et al. [45]. Black elderberry (BE) is one of the richest sources of anthocyanins, which improved some metabolic disturbances present in diet-induced obese C57BL/6J mouse model by lowering serum triglycerides (TAG), inflammatory markers, and IR [46].

Consequences caused by the intake of HF diet, such as hepatic steatosis, adipose hypertrophy, and IR, were attenuated by the intake of mulberry ethanol extract (MEE). Treatment with MEE altered the expression profile of genes involved in the lipid metabolism and acted as a protective mechanism on induced fatty acid oxidation, as well as it decreased fatty acid and cholesterol biosynthesis in vivo [47]. Wu et al. [48] reported that anthocyanins purified from Chinese mulberry (*Morus australis Poir*), such as cyanidin-3-glucoside, cyanidin-3-rutinoside, and pelargonidin-3-glucoside, after 12 weeks significantly inhibited body weight gain, reduced the IR, lowered the size of adipocytes, attenuated lipid accumulation, and decreased leptin secretion.

Goka fruit (GF), rich in anthocyanin, improved glucose tolerance and insulin sensitivity and reduced plasma insulin and hepatic accumulation of lipid in HF diet-induced obese mice after GF treatment for 12 weeks. In addition, GF administration to HF mice arose obesity-associated IR and hepatic lipid accumulation through the modulation of AMPK activity and lipid metabolism-associated gene expression [49]. Similar results were obtained by other authors with Cornelian cherries (*Cornus mas*) [50–52] and sweet orange (*Citrus sinensis* L. Osbeck) [53].

Hypolipidemic, antioxidant, and anti-inflammatory properties of blueberry (BB), blackberry (BK), and blackcurrant (BC) in a diet-induced obesity (DIO) mouse model were reported by Kim et al. [54]. Since BB, BK, and BC vary in the anthocyanin composition, their effect on plasma lipids and adipose macrophage infiltration in DIO mice was different. However, no differences were found in their antioxidant capacity and anti-inflammatory potential after BB, BK, and BC administration in DIO mice [55].

Boušová et al. [55] demonstrated that a cranberry extract- (CBE-) enriched diet differently modulated antioxidant enzymes and redox status in obese and nonobese mice. This study was designed to test the consumption of a CBE-enriched diet (2%) for 28 days and to compare the antioxidant status of nonobese and obese mice in a model with monosodium glutamate-induced obesity. CBE treatment increased the thiol content in the plasma and the glutathione S-transferase activity in the erythrocytes in both obese and nonobese mice. However, in the obese animals, the malondialdehyde (MDA) level in the erythrocytes was reduced, while hepatic NAD(P)H:quinone oxidoreductase and catalase activities in erythrocytes and small intestine were increased.

Anthocyanins can induce changes in adipose tissue, such as those in the expression levels of adipocytokines. The supplementation with black chokeberry (*Aronia melanocarpa*) juice concentrate (AJC) decreases epididymal fat (for −30%) and positively influences on adiponectin in male C57BL/6J mice. Therefore, this supplementation prevented weight gain and modulated markers of obesity [56]. Takahashi et al. [57] found that anthocyanin-rich Aronia fruits could suppress visceral fat accumulation and hyperglycemia through the inhibition of pancreatic lipase activity and thus lead to a reduction of intestinal lipid absorption in rats after feeding for 4 weeks.

Polyphenol-rich blackcurrant extract (BCE) prevented inflammation in male C57BL/6J mice fed with modified AIN-93M diet for 12 weeks [58]. The percentage of adipocyte size of the epididymal fat was lower than that in the control. The mRNA expression of genes involved in mitochondrial biogenesis, such as PPAR*α*, PPAR*δ*, UCP-2, UCP-3, and mitochondrial transcription factor A, were increased in the skeletal muscle in the group BCE-fed mice. Tumor necrosis factor *α* (TNF*α*) and interleukin-1*β* (IL-1*β*) mRNA were lower in splenocytes from BCE-fed mice than in the control in model-stimulated inflammation with lipopolysaccharides [58].

Gut microbes are important due to their possible involvement in the development of obesity process and chronic inflammation as well as IR [59]. In addition to being designed to reduce adiposity and hepatic lipogenesis in vivo, table grape consumption may alter gut microbiota. Sulfidogenic bacteria intestinal abundance was decreased, and the abundance of beneficial bacteria was increased in C57BL/6J mice with 3% grapes for 11 weeks. Obese mice fed with the grape polyphenol-rich diet had lower percentages of body fat and amounts of WAT and improved glucose tolerance compared to the HF-fed controls. In addition, these treatments with grape altered gut community structure and WAT inflammation [60].

## 4. The Role of Anthocyanins in Obesity: Human Studies

Despite the encouraging results obtained by preclinical studies in animal models, the role of anthocyanins in obesity deriving from clinical trials remains controversial. Few interesting reviews have been published in recent years on this topic, and all of them concluded that further interventional studies are needed to assess the preventive effects of anthocyanin-containing foods in obesity, diabetes, and metabolic syndrome due to the difficulty to establish the optimal dose and to identify the ideal food matrix for the best anthocyanin supplementation [27, 61]. Observational studies are more prone to suggest an antiobesity role of this class of polyphenols. In a recent work which analyzed food frequency questionnaires from about 124,000 participants from three different cohorts (Health Professionals Follow-up Study, Nurses' Health Study, and Nurses' Health Study II), the authors observed that an increased consumption of several flavonoid subclasses, including anthocyanins, was associated with weight loss in both men and women, aged 27–65, in a follow-up of 24 years. For example, in four years, increasing the daily consumption of BB by one half cup resulted in a weight loss of about 1.03 kg, less than 0.5 kg/year, a small but significant decrease potentially associated with health improvement [62]. Two interventional studies ended up with positive outcomes of blueberries supplementation in obese men and women at risk of cardiovascular disease and insulin resistance. In one case, 48 subjects, largely women, with metabolic syndrome and an average BMI of 37.8 ± 2.3 kg/m^2^, received for 8 weeks a blueberry beverage containing 50 g of freeze-dried blueberries (corresponding to 742 mg of total anthocyanins and 1624 mg total polyphenols). In the blueberry-supplemented group, systolic and diastolic blood pressures, plasma oxidized LDL, and serum malondialdehyde and hydroxynonenal concentrations decreased greater and significantly than in the control group, suggesting that blueberry beverage could contribute to ameliorate improve metabolic syndrome and related cardiovascular risk factors [63]. In the second study, 32 obese men and women with a BMI between 32 and 45 kg/m^2^ and insulin resistant received twice daily a smoothie with blueberry (45 g of powder contained 1462 mg of total polyphenolics of which 668 mg represented anthocyanins) for 6 weeks. The most interesting and significant change regraded insulin sensitivity which improved in the blueberry group (1.7 ± 0.5 mg·kg FFM^−1^·min^−1^) compared to the placebo group (0.4 ± 0.4 mg·kg FFM^−1^·min^−1^) [64].

In a different experimental approach, the administration of a so-called “gastrointestinal microbiome modulator” (GIMM) containing inulin, oat *β*-glucan, blueberry anthocyanins, and blueberry polyphenols in a small number overweight or obese individuals (30 subjects, age 18–70) reduced in four weeks the desire to eat in the GIMM group compared to placebo [65]. However, it is difficult to extrapolate from the data obtained which component(s) can be more directly responsible for the increased sense of satiety compared to the other measured outcomes, such as improved glucose tolerance. Supplementation with red orange juice rich in anthocyanins in 30 overweight healthy human volunteers for 12 weeks also resulted in a significant antiobesity effect measured as reduction in body mass index (BMI; 1.11 ± 0.09 kg/m^2^) compared to the placebo group (0.15 ± 0.09 kg/m^2^) [66].

Less encouraging results have been obtained in the QUENC trial, where 16 healthy volunteers aged 18–65 years with a BMI in the range of 25–35 kg/m^2^ consumed 3 sachets (8.3 g/sachet) per day of purple carrots for 4 weeks, providing a total daily intake of anthocyanin of 118.5 mg (259.2 mg total polyphenols/die). At the end of the trial, these obese subjects did not show any evidence of reduced body mass, and sense of appetite as well as changes in other markers of inflammation and lipid metabolism [67].

In the studies where other metabolic dysfunctions associated with obesity have been considered, such as metabolic syndrome, the supplementation with anthocyanin-enriched foods, for example, berry, in general, did not change weight or body composition [61] with references and tables therein. An exception is represented by a study where bilberry supplementation induced a decrease in weight and waist circumference in obese women [68]. Fluctuating results on the beneficial effects of anthocyanin supplementation in overweight and obese subjects and in individuals affected by metabolic syndrome have also been observed when other clinical parameters have been measured, such as diastolic and systolic blood pressure, dyslipidemia, blood glucose, and insulin resistance [27, 61, 69, 70]. To explain the alternate results observed in these studies, based on the supplementation of different berries (strawberry, cranberry, bilberry, sea buckthorn, blueberry, chokeberry, etc.), variations among the levels of anthocyanins in these berries can be easily hypothesized. It is not difficult to imagine that anthocyanin content changes quantitatively and qualitatively in distinct food sources. However, cause-effect relationships between chemical composition of anthocyanin supplements and their clinical outcomes is not easy to demonstrate and to approach experimentally. This goal may represent one of the challenges of this field in the next years.

The uncertainty of the clinical studies proving antiobesity effects of anthocyanins is confirmed by data summarized in Table 1. Here, we interrogated the http://ClinicalTrials.gov database, a service of the U.S. National Institutes of Health, for “anthocyanins” and “obesity” and retrieved a total of 16 studies (last update March 28, 2017). Those more closely related to the scope of this review have been reported in Table 1: all represent interventional studies and none of their results have been posted. It is easily to predict that, when and if results will be available, these trials will probably provide only limited breakthroughs in the field for the following reasons: (i) small number of enrolled subjects; (ii) wide age range in the enrolled subjects (young, adults, and elderly) which may generate confounding results due to the age-related differences in the physiopathology of obesity and related diseases; and (iii) wide differences in the type, composition, origin, doses, and duration of anthocyanins supplemented. The absence of standardized preparations of anthocyanins represents, in our opinion, an important confounding factor largely responsible for the variability and controversial results of clinical trials commented above. In fact, different typologies of anthocyanin formulations are generally employed in these studies (Table 1). In addition, another important aspect to be considered is the doses of anthocyanins necessary to reach the desired biological effects, which depend upon the complex pharmacokinetics of these compounds, as recently and exhaustively reviewed [36].

## 5. Conclusion

From the data reported above, it is easy to conclude that the potential functional relationship existing between anthocyanin supplementation and antiobesity effects suffers of the same pros and cons evidenced when the beneficial responses to other phytochemical treatments towards different degenerative diseases have been considered. In general, the positive and encouraging outcomes deriving from of preclinical studies (cell lines and animal models) are contradicted or, at least, diminished moving to case-control trials. The reasons behind this partial failure are complex and not easy to address. They certainly involve the different dosages applied in animal versus clinical studies, the complex metabolism and biotransformation to which anthocyanins and phytochemicals are subjected in the intestine and tissues, the possibility that different components present in the supplemented mixtures (e.g., berry extracts) can interact generating antagonistic, synergistic, or additive effects difficult to predict, and so on. However, we would like to highlight another issue which may contribute to generate confusion in the field, that is, the difference between prevention and therapy: prevention implies low doses, but long duration of the treatments (years), while therapy is associated with higher doses (and potentially side effects), but shorter time of administration. As an example, it is risky to hypothesize that anthocyanins can prevent obesity based on studies on already obese mice that received pharmacological doses of these phytochemicals. We believe that the evolution of the field must seriously consider the need to establish new and adequate cellular and animal models which may, in turn, allow the design of more efficient and prevention-targeted clinical studies.

## Figures and Tables

**Table 1:** Clinical studies retrieved from the http://ClinicalTrials.gov database on anthocyanins and obesity.

Trial number status	Conditions and dosage	Objectives	Primary outcome	Number of subjects (age/sex)
NCT02613715 (completed)	Overweight and obesity (250 ml of blackberry juice)	To evaluate the bioavailability of blackberry juice anthocyanins in normal weight and overweight/obese adults	Plasma concentrations of anthocyanins and anthocyanin metabolites	18 (18–40/M-F)
NCT01180712 (recruiting)	Type 2 diabetes (1.4 g of concentrated blackberry extract mirtoselect provided by Indena S.p.A.)	To determine the effects of anthocyanin supplementation in the form of a concentrated blackberry extract on insulin resistance and inflammation particularly in the adipose tissue	Oral glucose tolerance test	60 (40–70/M)
NCT01883401 (completed)	Dyslipidemia; obesity (25–50 g freeze-dried strawberries/day)	To determine the effects of low and high doses of freeze-dried strawberries in cardiovascular risk factors in subjects with abdominal adiposity and dyslipidemia	Change in lipids and lipoproteins	60 (19–72/M-F)
NCT01005420 (completed)	Insulin sensitivity (45 g of blueberry powder)	To evaluate the effect of blueberry powder on insulin sensitivity in obese, nondiabetic, and insulin-resistant subjects.	Insulin sensitivity	37 (>20/M-F)
NCT02689765 (completed)	Insulin resistance; glucose and lipid metabolism disorders; type 2 diabetes (80 g of a mixture of fresh blueberries and blackcurrants)	To characterize the potential effects of anthocyanins, purified from bilberries and blackcurrants, on metabolic zabnormalities commonly associated with type 2 prediabetes	Change in fasting glucose and HbA1C	160 (40–75/M-F)
NCT01705093 (unknown)	Childhood obesity; cardiovascular disease (50 g of flavonoid-rich freeze-dried strawberry powder)	To verify if strawberry intake can lead to improvements in select measures of cardiovascular function in overweight and obese adolescent males	Vascular function measured by peripheral arterial tonometry	25 (14–18/M)
NCT02035592 (active, but not recruiting)	Insulin resistance; metabolic syndrome X (13–26 g of freeze-dried blueberry powder per day)	To determine the dose-dependent impact of blueberry powder intake on insulin sensitivity and resistance, cardiovascular disease risk factors, and lung and cognitive function in overweight and obese participants with metabolic syndrome	Insulin resistance	144 (50–74/M-F)
NCT02291250 (recruiting)	Type 2 diabetes (200 g of blackcurrants, which contain anthocyanins, or 200 g greencurrants, which naturally contain no anthocyanins)	To investigate the acute effect blackcurrants on glucose metabolism in overweight/obese volunteers	Plasma glucose area under the curve	16 (21–70/M-F)
NCT02800967 (active, but not recruiting)	Hypertension; overweight (pure Aronia juice with appx. polyphenol content of 1000 mg gallic acid equivalents/100 ml)	To investigate the effects of Aronia juice polyphenols on platelet function and other CVD risk factors in subjects with moderate CVD risk	Changes in the percentage of P-selectin and glycoprotein IIbIIIa (GPIIbIIIa) positive platelets, percentage of platelet-monocyte, and platelet-neutrophil aggregates	84 (30–50/M-F)
NCT01564498 (recruiting)	Hypertension; hypercholesterolemia; type II diabetes; obesity; inflammation (300–500 g of cooked white/purple potatoes per day, or 300–500 g of raw orange/purple carrots per day)	To verify if purple vegetables, rich in polyphenolic compounds including anthocyanins, can have higher antioxidant and other biological activities than more lightly coloured versions of these foods	Blood cholesterol	60 (18–65/M-F)

## Abbreviations

Pg: Pelargonidin
Cy: Cyaniding
Pt: Petunidin
Pn: Peonidin
Dp: Delphinidin
MV: Malvidin
BMI: Body mass index
LDL: Low density lipoprotein
FFM: Fat mass and fat-free mass
GIMM: Gastrointestinal microbiome modulator
CPT1A: Carnitine palmitoyltransferase-1A
RBP4: Retinol binding protein-4
SREBP1c: Sterol regulatory element binding protein-1c
C/EBP*α*: CCAAT enhancer binding protein-*α*
PPAR *γ*: Peroxisomal proliferator-activated receptor gamma
AMPK: 5′ Adenosine monophosphate-activated protein kinase
GLUT4: Glucose transporter 4
GLUT2: Glucose transporter 2
ACC1: Acetyl-CoA carboxylase
FAS: Fatty acid synthase
LPL: lipoprotein lipase
PEPCK: Phosphoenolpyruvate carboxykinase
G6pase: Glucose-6-phosphatase
G6PDH: Glucose-6-phosphate dehydrogenase
VEGF: Vascular endothelial growth factor
ICAM-1: Intercellular adhesion molecule
JNK: c-Jun N-terminal kinase
TGF*β*1: Transforming growth factor-beta
IL-6: Interleukin-6
TNF-*α*: Tumor necrosis factor alpha
MCP-1: Monocyte-chemo-attractant protein-1.
